# J-Shaped Relationship Between Serum Prolactin and Metabolic-Associated Fatty Liver Disease in Female Patients With Type 2 Diabetes

**DOI:** 10.3389/fendo.2022.815995

**Published:** 2022-02-11

**Authors:** Cuiling Zhu, Huihui Ma, Dongdong Huang, Guifang Li, Jingyang Gao, Meili Cai, Hui You, Le Bu, Shen Qu

**Affiliations:** ^1^ Department of Endocrinology and Metabolism, Shanghai Tenth People’s Hospital, Tongji University School of Medicine, Shanghai, China; ^2^ National Metabolic Management Center, Shanghai Tenth People’s Hospital, Tongji University School of Medicine, Shanghai, China; ^3^ Department of Respiratory Medicine, Shanghai Pulmonary Hospital, Tongji University School of Medicine, Shanghai, China; ^4^ Department of Cardiology, People’s Hospital of Pu’er City, Pu’er, China

**Keywords:** prolactin, metabolic-associated fatty liver disease, type 2 diabetes, gender-specific difference, liver fibrosis

## Abstract

**Background:**

Metabolic-associated fatty liver disease (MAFLD) has become a worldwide epidemic. Prolactin (PRL), a pituitary hormone, has been linked to MAFLD. As a result, we set out to look into the relationship between serum PRL and the risk of MAFLD in patients with type 2 diabetes mellitus (T2DM).

**Methods:**

A total of 724 adults with T2DM were enrolled and categorized as MAFLD and non-MAFLD groups. Anthropometric data, biochemical parameters, and serum PRL levels were collected. Liver steatosis and fibrosis were assessed using FibroScan. Patients were stratified into normal PRL (NP) and high PRL (HP) groups and divided into four groups based on serum PRL quartiles. Multivariate logistic regression analysis was performed to evaluate the association between serum PRL and MAFLD risk.

**Results:**

Female but not male patients with MAFLD, liver steatosis, and fibrosis had significantly lower PRL levels in the NP group but higher PRL levels in the HP group than their counterparts. The proportions of MAFLD, liver steatosis, and fibrosis were significantly decreased in the NP group but increased in the HP group across the PRL quartiles in females but not in males. After multivariate adjustment, the adjusted ORs (AORs) and 95% CI for MAFLD among females were 18.165 (3.425–96.336), 1.784 (0.658–5.002), 1.744 (0.608–4.832), and 1.00 (reference) in the NP group (Q1–Q4, *P*-trend *<* 0.001) and 1.00 (reference), 11.098 (1.819–110.356), 15.225 (1.996–116.112), and 18.211 (2.579–128.568) in the HP group (Q1–Q4, *P*-trend = 0.020). Such associations were also found between serum PRL and liver fibrosis in females but not in males.

**Conclusion:**

We observed a J-shaped association between serum PRL and the risk of MAFLD and liver fibrosis in females but not in males with T2DM, indicating that PRL may be relevant to MAFLD and its progression in a gender-specific manner.

**Clinical Trial Registration:**

Chinese Clinical Trial Registry, number ChiCTR-OCS-12002381.

## Introduction

Metabolic-associated fatty liver disease (MAFLD) is a new definition of non-alcoholic fatty liver disease (NAFLD) and is mainly defined as liver fat deposition along with obesity, diabetes, or combined metabolic disorders (1, 2). This change emphasizes the importance of metabolic disorder complicated with fatty liver regardless of the heterogeneous etiology since the risk of MAFLD for significant fibrosis, cirrhosis, and mortality is largely attributable to its metabolic disorders (3, 4). MAFLD is also regarded as the hepatic manifestation of multisystem metabolic dysfunction (5), and available studies have suggested that patients with MAFLD were more likely to have worse metabolic profiles than NAFLD (6). Therefore, MAFLD is believed to be superior to the NAFLD definition for predicting metabolic at-risk patients. MAFLD and type 2 diabetes mellitus (T2DM) are common conditions that generally coexist and share insulin resistance (IR) as a major pathophysiological mechanism. In the meta-analysis of Younossi et al. (7), the global prevalence of NAFLD in individuals with T2DM is 55.5%, two-fold higher than that in the general population. An interesting study by Bril et al. (8) also revealed that NAFLD is increasingly common in patients with T2DM, whose estimated prevalence is between 60% and 80%. Conversely, NAFLD is associated with an almost two-fold increased risk of incident T2DM (9). Considering that MAFLD and T2DM have synergistic effect on driving multiple complications, including cardiovascular disease, liver-related mortality, and all-cause mortality (3, 6, 10), therefore, identifying the risk factors and possible pathogenesis of MAFLD and evaluating liver fibrosis in the diabetic population are critical for early detection and management of MAFLD patients.

To our notice, serum prolactin (PRL) has been previously reported to be associated with NAFLD; however, the role of PRL in the development and progression of MAFLD has not yet been studied. PRL, a multifunctional pituitary hormone produced predominantly by the anterior pituitary gland (11), involves diverse biological functions, including reproduction and lactation, osmoregulation, immune modulation, and metabolic homeostasis (12). Among them, the role of PRL in glucolipid metabolism has been the focus of research in recent years (13). In population-based studies, a high-normal serum PRL within the normal physiological range is associated with a lower risk of T2DM (14) and improved visceral fat dysfunction and IR (15) and negatively associated with NAFLD and severity of hepatic steatosis in both men and women (16). Furthermore, in experimental studies, PRL was demonstrated to protect against gestational diabetes (17), improve insulin sensitivity in obese males (18), and attenuate hepatic fat accumulation in female mice (19). However, it is worth noting that hyperprolactinemia outside the normal physiological range has been reported to have an increased risk of renal disease, hypogonadism, hypothyroidism, and PCOS, as well as cardiovascular and all-cause mortality (20–23). Thus, the relationship between PRL and metabolic disorders is complex and varies depending on whether serum PRL is within or outside the physiological range.

Although a close relationship between serum PRL and hepatic lipid accumulation is generally accepted, little is known about the correlation of serum PRL with the risk of incident MAFLD and liver fibrosis in individuals with T2DM. Moreover, previous studies did not evaluate their association in males and females based on serum PRL levels within or outside the normal reference range separately. Based on the significant advantage of MAFLD to NAFLD with regard to metabolic assessment, therefore, the purpose of this study was to examine the association between serum PRL levels and the occurrence of MAFLD and liver fibrosis in a population with T2DM stratified by different PRL subgroups and genders, aiming to provide novel insights into the correlation between serum PRL and the severity of MAFLD within a similar population.

## Materials and Methods

### Study Design and Participants

**Figure 1:**
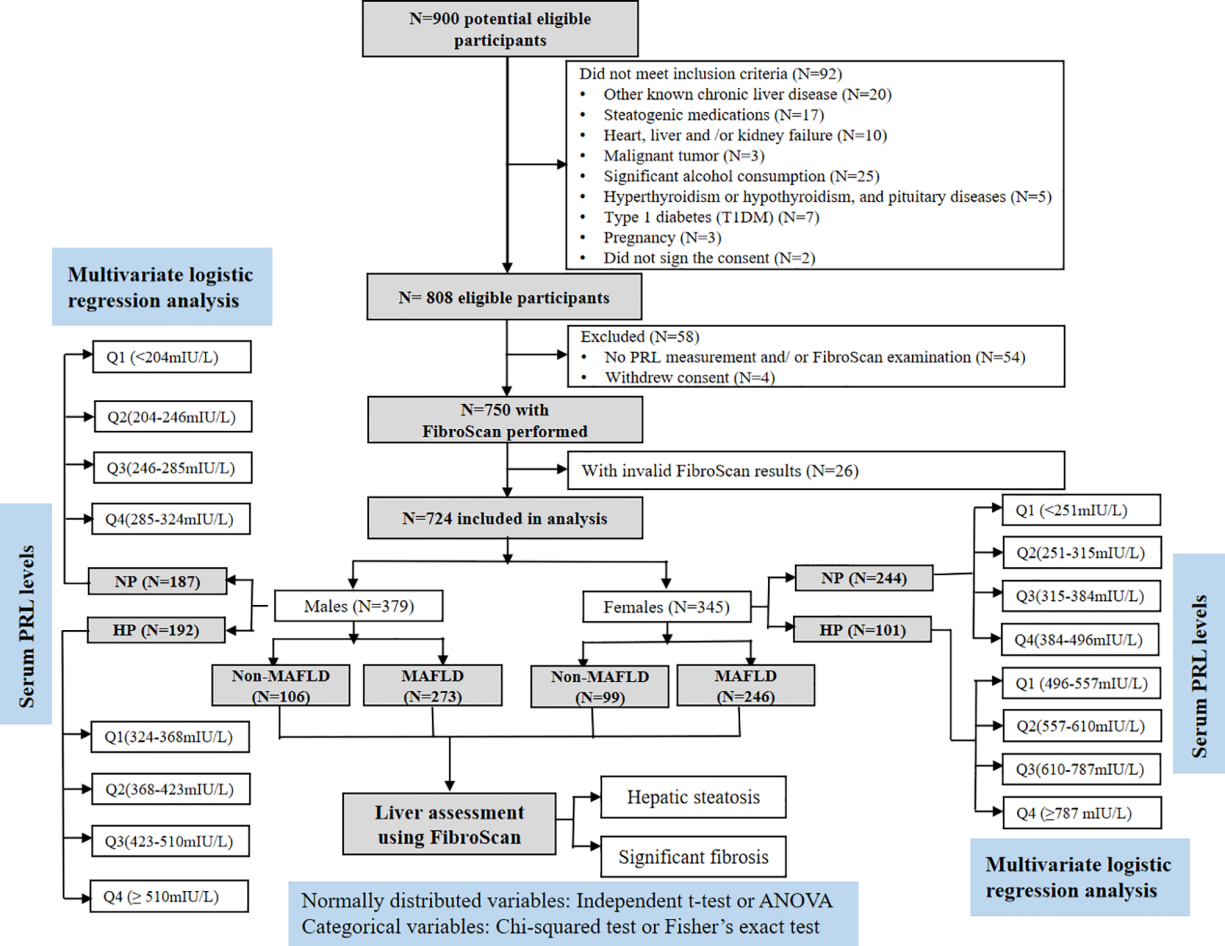
Flowchart of the study enrollment. Of the 900 selected patients, those who did not meet the inclusion criteria (*n* = 146) or decide to withdraw from the study (*n* = 4) were excluded. Another 26 patients with invalid FibroScan results were also excluded. As a result, 724 patients were included in the final analysis.

This retrospective cross-sectional study was conducted at the Department of Endocrinology and Metabolism, Shanghai Tenth People’s Hospital in China between January 2017 and February 2021. A total of 900 patients with T2DM who met the following inclusion criteria were consecutively enrolled: 1) aged 18~65 years old and (2) underwent laboratory tests, hepatic ultrasonography, and valid transient elastography (FibroScan) examination. Exclusion criteria included patients with the presence of 1) other known chronic liver diseases, such as chronic hepatitis B or C, autoimmune hepatitis, and hemochromatosis; 2) pre-existing active cancer, renal dysfunction, severe liver dysfunction, congestive heart failure, or free abdominal fluid; 3) history of hyperthyroidism or hypothyroidism, pituitary diseases, and other types of diabetes, 4) significant alcohol consumption (24); 5) pregnancy; and 6) receiving any therapeutic methods that could lead to liver steatosis or fibrosis and influence the glucolipid metabolism or PRL levels, such as lipid-lowering and PRL-lowering agents (bromocriptine), as well as chlorpromazine and other phenothiazines, haloperidol, tricyclic antisuppressants, olanzapine, high-dose estrogen, some antihistamines, α-methyl-dopamine, synthetic TRH, anesthetics, arginine, and insulin injection within 6 months prior to this study. Finally, 724 subjects (379 males and 345 females) were analyzed in this study, as shown in **Figure 1**.

### Clinical and Biochemical Measurements

Demographic and clinical information, including age, sex, height, body weight, waist circumference (WC), hip circumference (HC), systolic blood pressure (SBP), diastolic blood pressure (DBP), lifestyle factors (smoking status and alcohol consumption), comorbidities, and medical history, was collected by trained physicians. BMI was calculated as weight in kilograms divided by height in meters squared. After a 12-h overnight fast, all participants had their venous blood drawn in the morning. Alanine transaminase (ALT), aspartate aminotransferase (AST), gamma-glutamyl transferase (γ-GT), total cholesterol (TC), triglycerides (TG), low-density lipoprotein cholesterol (LDL-C), high-density lipoprotein cholesterol (HDL-C), fasting plasma glucose (FPG), fasting insulin (FINS), glycosylated hemoglobin (HbA1c), C-reactive protein (CRP), progesterone (Pg), estradiol (E2), and total testosterone (TT) were measured. PRL levels were measured by electrochemiluminescent immunoassay. Homeostasis model assessment of IR (HOMA-IR) was calculated as described by Matthews et al. (25): FPG (mmol/L) × FINS (mU/L)/22.5. All the laboratory measurements were conducted in our department using standard methodologies.

### Diagnosis and Subgroup

MAFLD was diagnosed based on evidence of ultrasonically diagnosed hepatic steatosis in addition to one of the three criteria proposed by the international expert consensus statement in 2020, namely, overweight/obesity, T2DM, or metabolic dysregulation regardless of alcohol consumption or other concomitant liver diseases (2). Herein, metabolic dysregulation was defined by the presence of at least two metabolic risk abnormalities found in lean or normal weight patients, including hypertension, dyslipidemia, hyperglycemia, IR, and high CRP levels. In Asians, overweight or obesity was defined as BMI ≥23 kg/m^2^. Abdominal obesity was diagnosed when WC ≥90/80 cm in Asian men and women. Hypertension was defined by blood pressure ≥130/85 mmHg or the use of antihypertensive drugs. Dyslipidemia was defined by plasma TG ≥1.7 mmol/L in the total population or plasma HDL‐C <1.0 mmol/L for men and <1.3 mmol/L for women or by specific drug treatment. T2DM was diagnosed according to the guideline for the prevention and treatment of T2DM in China (2020 edition) (26). In addition, plasma CRP >2 mg/L or HOMA-IR ≥2.5 was also regarded as MAFLD-associated metabolic abnormalities. Finally, patients were categorized as MAFLD (*n* = 519) and non-MAFLD groups (*n* = 205) based on hepatic ultrasonography and biochemical tests.

Due to significant gender difference in serum PRL levels, we then separated subjects into the following subgroups: normal PRL (NP) group (males, *n* = 187; females, *n* = 244) and high PRL (HP) group (males, *n* = 192; females, *n* = 101). Herein, HP was defined as serum PRL ≥324 mIU/L in males or ≥496 mIU/L in females according to the normal reference value of serum PRL in our hospital. Conversely, NP was defined as serum PRL <324 mIU/L in males or <496 mIU/L in females. Furthermore, subjects were divided into four quartiles (Q1, Q2, Q3, and Q4) according to the serum PRL levels: in the NP group among males, Q1: <204 mIU/L, Q2: 204–246 mIU/L, Q3: 246–285 mIU/L, and Q4: 285–324 mIU/L; in the HP group among males, Q1: 324–368 mIU/L, Q2: 368–423 mIU/L, Q3: 423–510 mIU/L, and Q4: ≥510 mIU/L; in the NP group among females, Q1: <251 mIU/L, Q2: 251–315 mIU/L, Q3: 315–384 mIU/L, and Q4: 384–496 mIU/L; and in the HP group among females, Q1: 496–557 mIU/L, Q2: 557–610 mIU/L, Q3: 610–787 mIU/L, and Q4: ≥787 mIU/L.

### Liver Ultrasound and Transient Elastography (FibroScan)

Hepatic steatosis in this study was detected by hepatic ultrasonography according to specific imaging features, such as diffuse hyperechogenicity of liver–kidney contrast, ultrasound beam attenuation, and intrahepatic vascular blurring. In addition, controlled attenuation parameter (CAP) and liver stiffness measurement (LSM) were obtained from transient elastography (FibroScan^®^) using the M probe or the XL probe, according to the recommendation by the software. FibroScan was considered successful when there were at least 10 valid measurements with measurement variability <30% of the mean (27). According to literature consensus, a patient was considered to have hepatic steatosis if CAP value ≥248 dB/m or to have significant hepatic fibrosis if LSM ≥7.0 and ≥6.2 kPa (using either M or XL probes) (28). The hepatic ultrasonography and transient elastography were performed and evaluated by experienced ultrasonographers blinded to the clinical and biochemical details of the participants.

### Statistical Analysis

All the statistical analyses were performed using SPSS 23.0 software, and the figures in this study were produced by GraphPad Prism 6.0 project. Data were firstly tested for normal distribution with the Kolmogorov–Smirnov test. Continuous variables were expressed as means ± standard deviation (SD) or medians (interquartile ranges), and categorical variables as absolute and relative proportions (*n*, %). Independent Student’s *t*-test or one-way analysis of variance (ANOVA) for normally distributed variables was tested to compare the variables between groups. Non-normally distributed data were analyzed by non-parametric test. Categorical variables were analyzed using the chi-squared test or Fisher’s exact test when appropriate. Non-normally distributed data were logarithmically transformed to normality, when needed. Linear regression analysis was used to determine the relationship between serum PRL and MAFLD-related metabolic risk factors in males and females. Moreover, binary and multivariate logistic regression analysis was performed to investigate the correlation between serum PRL and the occurrence of MAFLD and significant liver fibrosis in three different models: 1) for age and BMI; 2) for age, BMI, CAP, LSM, ALT, AST, and γ-GT; and 3) for HDL-C, TG, FPG, E2, TT, and Pg in addition to all covariates in (2) both in females and males. Odds ratios (ORs) and 95% confidence intervals (95% CI) were calculated. A two‐tailed *P <*0.05 was considered to be statistically significant.

## Results

### Baseline Clinical and Metabolic Characteristics of Study Participants

As shown in **Table 1**, the baseline clinical and metabolic characteristics of 724 diabetic patients were assessed, stratified by MAFLD status and gender. The mean age of the study population was 47.7 ± 17.2 years, and 52.3% were males (*n* = 379). Based on the 2020 international expert consensus statement (1), 519 (71.7%) of patients were diagnosed with MAFLD. Moreover, 593 (81.9%) and 323 (44.6%) of the total cohort had liver steatosis and significant fibrosis determined by FibroScan. Compared with patients without MAFLD, those with MAFLD were younger and had significantly higher BMI, WC, DBP, ALT, AST, γ-GT, TG, CAP, LSM, LnFINS, LnHOMA-IR, and CRP levels as well as lower HDL-C levels in both genders (all *P* < 0.01). Also, significantly increased SBP and FPG levels were observed in males but not in females with MAFLD, in comparison to their counterparts. Additionally, male patients with MAFLD had significantly higher Pg levels (*P* < 0.001) but lower TT levels (*P* = 0.033) than those without MAFLD. Conversely, female patients with MAFLD had significantly higher TT levels (*P* = 0.011) but lower E2 levels (*P* = 0.003) than their counterparts. Furthermore, the proportions of overweight/obese, abdominally obese, hypertension, hyperlipidemia, and IR in the MAFLD group were significantly higher than those in the non-MAFLD group (all *P* < 0.05). As for liver assessment, subjects with MAFLD were more likely to have liver steatosis (89.0% vs. 53.8% in males, *P* < 0.001; 89.0% vs. 74.7% in females, *P* = 0.001) and significant fibrosis (47.3% vs. 20.8% in males, *P* < 0.001; 58.5% vs. 38.3% in females, *P* < 0.001) compared with those without MAFLD.

**Table 1:** Baseline metabolic characteristics of the study cohort stratified by MAFLD status in both genders.

Parameters	Males (*n* = 379)			Females (*n* = 345)		
	Non-MAFLD	MAFLD	*P-*value	Non-MAFLD	MAFLD	*P-*value
	(*n* = 106)	(*n* = 273)		(*n* = 99)	(*n* = 246)	
Demographics						
Age (years)	55.6 ± 10.5	44.8 ± 17.6	<0.001	53.7 ± 14.2	45.1 ± 18.3	<0.001
Anthropometrics						
BMI (kg/m^2^)	23.6 ± 3.4	31.5 ± 7.9	<0.001	26.1 ± 5.7	31.5 ± 7.4	<0.001
WC (cm)	90.8 ± 11.8	106.0 ± 19.1	<0.001	89.2 ± 15.1	99.7 ± 17.1	<0.001
SBP (mmHg)	131 ± 19	137 ± 17	0.004	137 ± 20	136 ± 18	0.752
DBP (mmHg)	74 ± 11	79 ± 13	<0.001	73 ± 11	78 ± 12	0.002
FibroScan						
CAP (dB/m)	243.1 ± 58.7	322.2 ± 57.6	<0.001	305.4 ± 84.5	354.3 ± 88.9	<0.001
LSM (kPa)	5.7 ± 2.6	7.7 ± 4.1	<0.001	6.7 ± 3.2	8.8 ± 4.1	<0.001
Laboratory parameters						
ALT (U/L)	18.7 (22.1)	30.0 (39.0)	<0.001	15.4 (19.7)	28.0 (39.2)	<0.001
AST (U/L)	15.9 (10.1)	22.2 (16.7)	<0.001	16.7 (11.4)	23.9 (21.6)	<0.001
γ-GT (U/L)	20.3 (22.5)	35.6 (39.4)	<0.001	17.7 (20.2)	30.3 (27.3)	<0.001
TC (mmol/L)	4.4 ± 0.9	4.6 ± 1.1	0.242	4.8 ± 1.1	4.8 ± 1.2	0.739
TG (mmol/L)	1.4 ± 0.9	2.3 ± 1.7	<0.001	1.7 ± 0.8	2.1 ± 1.1	0.013
HDL-C (mmol/L)	1.2 ± 0.4	1.0 ± 0.4	<0.001	1.2 ± 0.3	1.0 ± 0.3	0.036
LDL-C (mmol/L)	2.6 ± 0.8	2.7 ± 0.9	0.941	2.9 ± 0.9	2.8 ± 0.9	0.318
FPG (mmol/L)	7.3 ± 1.8	8.1 ± 2.9	0.002	7.7 ± 2.9	7.9 ± 2.9	0.596
LnFINS (mU/L)	1.6 ± 1.2	2.7 ± 1.0	<0.001	2.4 ± 1.1	2.9 ± 0.9	<0.001
LnHOMA-IR	1.3 ± 1.2	1.6 ± 0.9	<0.001	1.3 ± 1.1	1.7 ± 0.9	0.001
CRP (mg/L)	3.2 ± 1.1	5.2 ± 4.1	<0.001	3.2 ± 0.8	4.0 ± 1.4	<0.001
TT (nmol/L)	16.5 ± 6.5	10.5 ± 6.2	0.033	0.8 ± 0.7	2.0 ± 1.1	0.011
E2 (pmol/L)	101.2 ± 44.3	110.1 ± 49.1	0.149	144.4 ± 54.5	103.4 ± 41.6	0.003
Pg (nmol/L)	0.8 ± 0.1	2.9 ± 0.4	<0.001	2.7 ± 0.1	2.9 ± 0.4	0.553
Coexisting disorders, *n* (%)						
Overweight/obesity	58 (54.7)	248 (90.8)	<0.001	63 (63.6)	224 (91.1)	<0.001
Abdominal obesity	39 (36.8)	206 (75.5)	<0.001	56 (56.6)	213 (86.6)	<0.001
Hypertension	59 (55.7)	190 (69.6)	0.012	60 (60.6)	178 (72.7)	0.039
Hyperlipidemia	35 (34.0)	138 (52.5)	0.002	63 (64.3)	184 (76.0)	0.032
Insulin resistance	30 (28.3)	199 (73.2)	<0.001	62 (63.3)	197 (86.0)	<0.001
Liver assessment, *n* (%)						
Hepatic steatosis	57 (53.8)	243 (89.0)	<0.001	74 (74.7)	219 (89.0)	0.001
Significant fibrosis	22 (20.8)	129 (47.3)	<0.001	28 (38.3)	144 (58.5)	<0.001

Continuous data are presented as means ± standard deviations (SD) or medians (interquartile ranges). Non-normally distributed data were log-transformed before analysis. Categorical variables are presented as percentages (%). BMI, body mass index; WC, waist circumference; SBP, systolic blood pressure; DBP, diastolic blood pressure; CAP, controlled attenuation parameter; LSM, liver stiffness measurement; ALT, alanine transaminase; AST, aspartate aminotransferase; γ-GT, gamma-glutamyl transferase; TC, total cholesterol; TG, triglyceride; HDL-C, high-density lipoprotein cholesterol; LDL-C, low-density lipoprotein cholesterol; FPG, fasting plasma glucose; FINS, fasting insulin; HOMA-IR, homeostasis model assessment of insulin resistance; CRP, C-reactive protein. TT, total testosterone; E2, estradiol; Pg, Progesterone. P-values <0.05 were accepted as statistically significant.

### Percentage of MAFLD, Liver Steatosis, and Significant Fibrosis in Patients With T2DM

**Figure 2:**
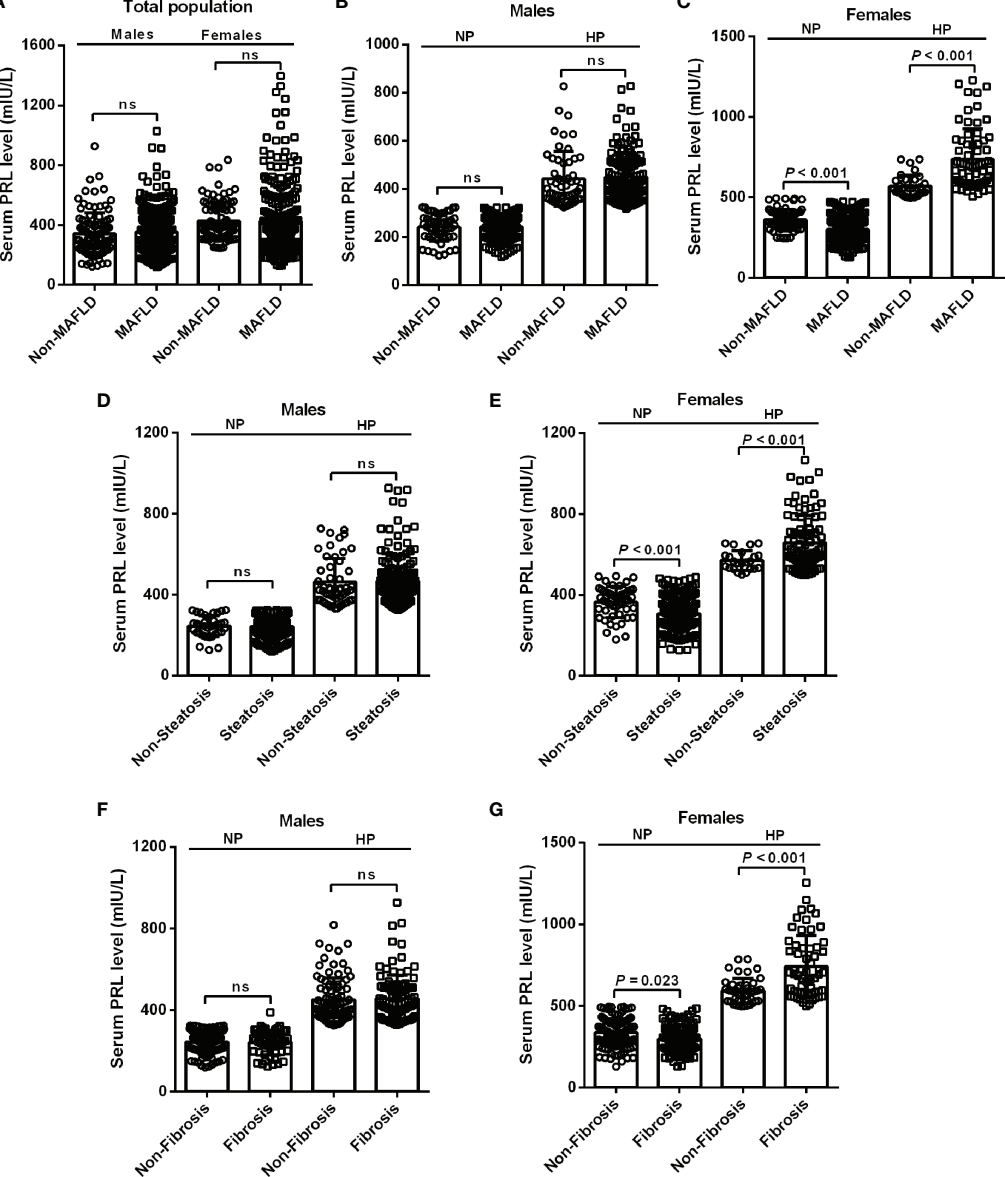
Levels of serum PRL among diabetic patients with or without MAFLD, steatosis, and fibrosis stratified by PRL levels and gender. **(A)** The serum PRL levels did not change significantly between the MAFLD and non-MAFLD groups in the total population. **(B, C)** Among females, patients with MAFLD had significantly decreased serum PRL levels within the NP group but increased serum PRL levels within the HP group compared with their counterparts, which was not observed in males. **(E, G)** Female patients with steatosis or fibrosis had significantly lower serum PRL levels within the NP group but higher serum PRL levels within the HP group compared with their counterparts. **(D, F)** No significant differences were observed in serum PRL levels among males with steatosis or fibrosis compared with their counterparts neither in the NP group nor in the HP group. The high PRL (HP) group was defined as serum PRL ≥324 mIU/L in males or ≥496 mIU/L in females. The normal PRL (NP) group was defined as serum PRL <324 mIU/L in males or <496 mIU/L in females. PRL, prolactin. *P*-values <0.05 were accepted as statistically significant. ns, non-significant.

**Figure 3:**
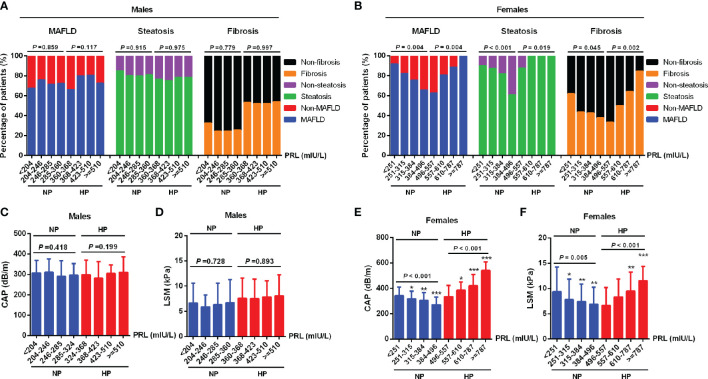
Percentage of MAFLD, liver steatosis, and fibrosis across serum PRL levels in diabetic patients. PRL levels were plotted into four quartiles in both genders. **(A)** Among males, the percentage of MAFLD, liver steatosis, and fibrosis did not change significantly across serum PRL quartiles. **(B)** Among females, there was a significantly decreasing trend in the percentage of MAFLD, liver steatosis, and fibrosis in the NP group but an increasing trend in the HP group across serum PRL quartiles. **(C, D)** CAP and LSM values did not change significantly across serum PRL quartiles among males. **(E, F)** Among females, CAP and LSM values were significantly decreased in the NP group but increased in the HP group across serum PRL quartiles. CAP, controlled attenuation parameter; LSM, liver stiffness measurement. *P*-values <0.05 were accepted as statistically significant. *P < 0.05, **P < 0.01, ***P < 0.001.

To investigate the relationship between serum PRL and MAFLD and hepatic fibrosis in type 2 diabetic patients, we divided patients into NP and HP groups. Intriguingly, female patients with MAFLD showed significantly decreased serum PRL levels in the NP group but increased serum PRL levels in the HP group (all *P* < 0.001) compared with those without MAFLD (**Figure 2C**), which was not observed among males and the total population (**Figures 2A, B**). Likewise, females with hepatic steatosis or significant fibrosis presented remarkably lower serum PRL levels in the NP group (*P* < 0.001, *P* = 0.023, respectively) but higher serum PRL levels in the HP group (all *P* < 0.001) as opposed to their counterparts (**Figures 2E, G**), which was not shown among males (**Figures 2D, F**). To explore the correlation of serum PRL with the development and progression of MAFLD, we divided the patients into four groups based on serum PRL quartiles. Notably, among females, there was a significantly decreasing trend in the percentage of MAFLD, liver steatosis, and fibrosis in the NP group (*P* = 0.004, *P* < 0.001, *P* = 0.045, respectively) but increasing trend in the HP group (*P* = 0.004, *P* = 0.019, *P* = 0.002, respectively) across serum PRL quartiles (**Figure 3B**), whereas such significant association was not shown among males (**Figure 3A**). A similar changing trend was shown in the CAP and LSM value with the increment of serum PRL in females (all *P* < 0.01) (**Figures 3E, F**) but not in males (**Figures 3C, D**).

### Correlation Between PRL and MAFLD-Related Metabolic Parameters in Patients With T2DM

**Figure 4:**
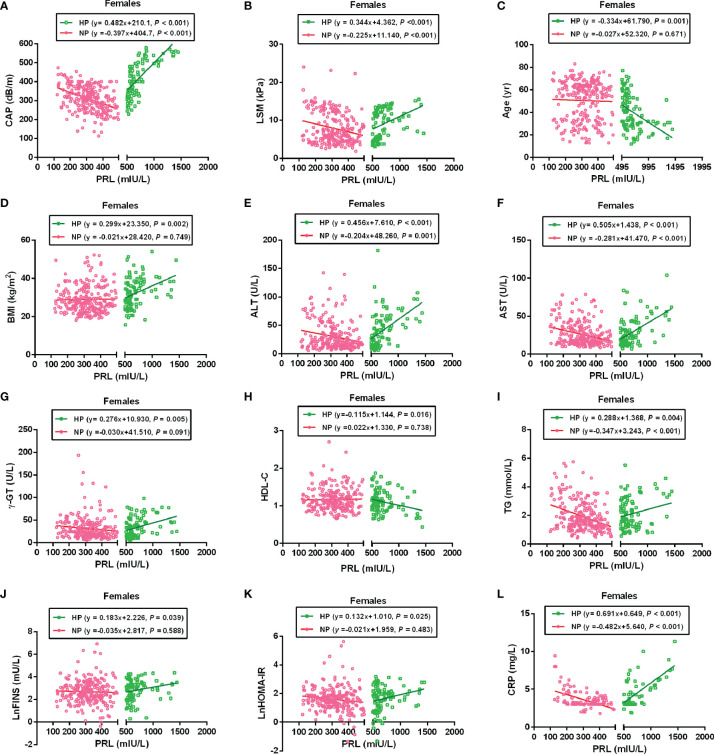
Linear regression analysis shows the relationship between PRL and MAFLD-related risk factors among females. These factors included CAP **(A)**, LSM **(B)**, Age **(C)**, BMI **(D)**, ALT **(E)**, AST **(F)**, γ-GT **(G)**, HDL-C **(H)**, TG **(I)**, LnFINS **(J)**, LnHOMA-IR **(K)**, and CRP **(L)**. In the NP group, serum PRL was significantly negatively associated with CAP, LSM, ALT, AST, TG, and CRP but not with age, BMI, γ-GT, HDL-C, LnFINS, and LnHOMA-IR. In the HP group, serum PRL was significantly positively associated with CAP, LSM, BMI, ALT, AST, γ-GT, TG, LnFINS, LnHOMA-IR, and CRP but negatively associated with age and HDL-C. PRL, prolactin; CAP, controlled attenuation parameter; LSM, liver stiffness measurement; ALT, alanine transaminase; AST, aspartate aminotransferase; γ-GT, gamma-glutamyl transferase; HDL-C, high-density lipoprotein cholesterol; TG, triglyceride; FINS, fasting insulin; HOMA-IR, homeostasis model assessment of insulin resistance; CRP, C-reactive protein. Non-normally distributed data were log-transformed before analysis. *P*-values < 0.05 were accepted as statistically significant.

**Figure 5:**
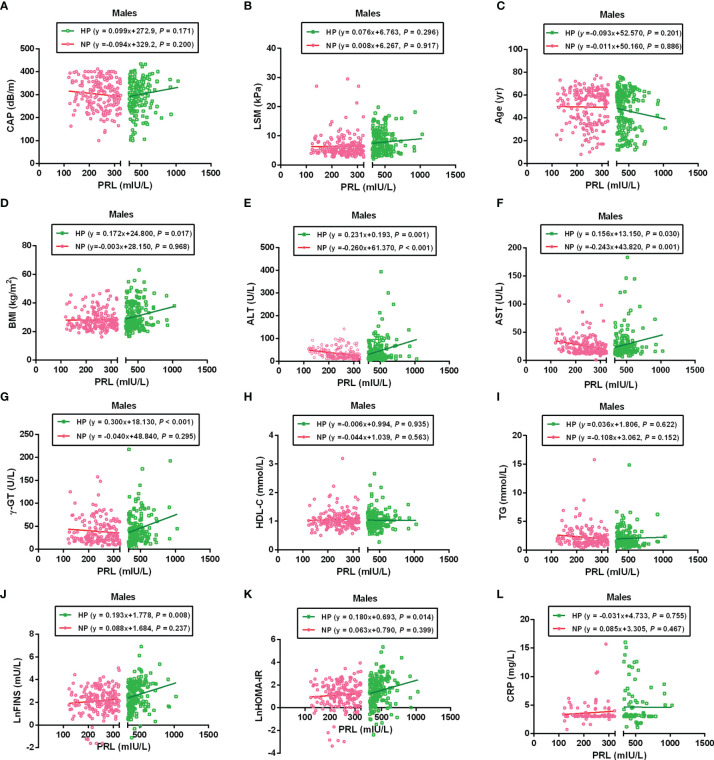
Linear regression analysis shows the relationship between PRL and MAFLD-related risk factors among males. These factors included CAP **(A)**, LSM **(B)**, Age **(C)**, BMI **(D)**, ALT **(E)**, AST **(F)**, γ-GT **(G)**, HDL-C **(H)**, TG **(I)**, LnFINS **(J)**, LnHOMA-IR **(K)**, and CRP **(L)**. The serum PRL was significantly negatively associated with ALT and AST in the NP group but positively associated with BMI, ALT, AST, γ-GT, LnFINS, and LnHOMA-IR in the HP group. PRL, prolactin; CAP, controlled attenuation parameter; LSM, liver stiffness measurement; ALT, alanine transaminase; AST, aspartate aminotransferase; γ-GT, gamma-glutamyl transferase; HDL-C, high-density lipoprotein cholesterol; TG, triglyceride; FINS, fasting insulin; HOMA-IR, homeostasis model assessment of insulin resistance; CRP, C-reactive protein. Non-normally distributed data were log-transformed before analysis. *P*-values < 0.05 were accepted as statistically significant.

To further analyze the association between PRL and MAFLD-related metabolic parameters in patients with T2DM, univariate and multivariate linear regression analyses were performed. Serum PRL levels in females were significantly inversely related to CAP, LSM, ALT, AST, TG, and CRP levels in the NP group but significantly positively related to CAP, LSM, BMI, ALT, AST, γ-GT, TG, LnFINS, LnHOMA-IR, and CRP levels in the HP group. In addition, serum PRL levels were negatively associated with age and HDL-C in the HP but not in the NP group among females (**Figure 4**). Among males, serum PRL levels were significantly negatively associated with ALT and AST levels in the NP group but positively associated with BMI, ALT, AST, γ-GT, LnFINS, and LnHOMA-IR levels in the HP group. No significant association was observed between PRL and CAP, LSM, age, HDL-C, TG, and CRP (all *P* > 0.05) either in the NP group or in the HP group among males (**Figure 5**). After adjusting for age and BMI, multivariate linear regression analysis showed that among females, serum PRL levels were significantly negatively correlated with CAP (*β* = −0.402, *P* < 0.001), LSM (*β* = −0.176, *P* = 0.031), AST (*β* = 0.229, *P* = 0.022), TG (*β* = −0.268, *P* < 0.001), and CRP (*β* = −0.343, *P* = 0.001) in the NP group but positively correlated with CAP (*β* = 0.307, *P* = 0.017), LSM (*β* = 0.262, *P* = 0.048), AST (*β* = 0.362, *P* = 0.012), TG (*β* = 0.287, *P* = 0.005), FPG (*β* = 0.851, *P* = 0.032), LnFINS (*β* = 0.118, *P* = 0.025), LnHOMA-IR (*β* = 0.386, *P* = 0.017), CRP (*β* = 0.432, *P* = 0.003), and TT (*β* = 0.261, *P* < 0.001) in the HP group. Among males, serum PRL levels were significantly negatively related to ALT (*β* = −0.377, *P* = 0.004) in the NP group but positively related to ALT (*β* = 0.498, *P* = 0.007) and γ-GT (*β* = 0.309, *P* < 0.001) in the HP group (**Table 2**).

**Table 2:** Multiple regression analysis showing the relationship between PRL and MAFLD-related risk factors in the NP and HP groups in both genders.

Independent variables	Males						Females					
	NP (*n* = 187)			HP (*n* = 192)			NP (*n* = 244)			HP (*n* = 101)		
	*β*	*t*	*P*	*β*	*t*	*P*	*β*	*t*	*P*	*β*	*T*	*P*
ALT (U/L)	−0.377	−2.942	0.004	0.498	2.727	0.007	0.088	0.832	0.406	0.116	0.850	0.398
AST (U/L)	−0.142	−1.174	0.242	−0.409	−2.274	0.054	−0.229	−2.303	0.022	0.322	2.568	0.012
γ-GT (U/L)	0.142	1.642	0.103	0.309	3.797	<0.001	0.123	1.928	0.055	-0.035	-0.323	0.748
TG (mmol/L)	−0.159	−1.819	0.071	0.011	0.127	0.899	−0.268	−4.044	<0.001	0.287	2.874	0.005
HDL-C (mmol/L)	−0.001	−0.011	0.992	0.024	0.316	0.752	0.150	−2.236	0.056	0.083	0.849	0.398
FPG (mmol/L)	0.08	0.233	0.816	0.175	0.474	0.636	0.155	0.546	0.586	0.851	2.183	0.032
LnFINS (mU/L)	0.788	0.668	0.506	0.812	0.564	0.574	0.339	0.406	0.685	0.118	2.293	0.025
LnHOMA-IR	−0.571	−0.477	0.634	−0.770	−0.536	0.593	−0.338	−0.407	0.684	0.386	2.439	0.017
CRP (mg/L)	−0.161	−0.237	0.334	−0.350	−0.332	0.333	−0.343	−0.411	0.001	0.432	2.212	0.003
TT (nmol/L)	0.133	1.313	0.191	−0.007	−0.060	0.952	0.056	0.753	0.452	0.261	2.674	<0.001
E2 (pmol/L)	−0.059	−0.638	0.524	−0.058	−0.641	0.522	−0.005	−0.062	0.951	0.016	0.170	0.865
Pg (nmol/L)	0.015	0.175	0.862	−0.094	−1.170	0.244	−0.061	−0.865	0.388	0.084	0.058	0.502
CAP (dB/m)	−0.024	−0.232	0.817	0.060	0.616	0.539	−0.402	−5.728	<0.001	0.307	2.441	0.017
LSM (kPa)	−0.045	−0.501	0.617	−0.086	−0.912	0.363	−0.176	−2.168	0.031	0.262	1.458	0.048

Multiple linear regression analysis was performed to analyze the relationships between PRL and MAFLD-related factors. P-values <0.05 were accepted as statistically significant. All the data were adjusted for age and BMI.

ALT, alanine transaminase; AST, aspartate aminotransferase; γ-GT, gamma-glutamyl transferase; TG, triglyceride; HDL-C, high-density lipoprotein cholesterol; FPG, fasting plasma glucose; FINS, fasting insulin; HOMA-IR, homeostasis model assessment of insulin resistance; CRP, C-reactive protein; TT, total testosterone; E2, estradiol; Pg, progesterone; CAP, controlled attenuation parameter; LSM, liver stiffness measurement.

### Influence of PRL on the Risk of MAFLD and Hepatic Fibrosis in Patients With T2DM

**Figure 6:**
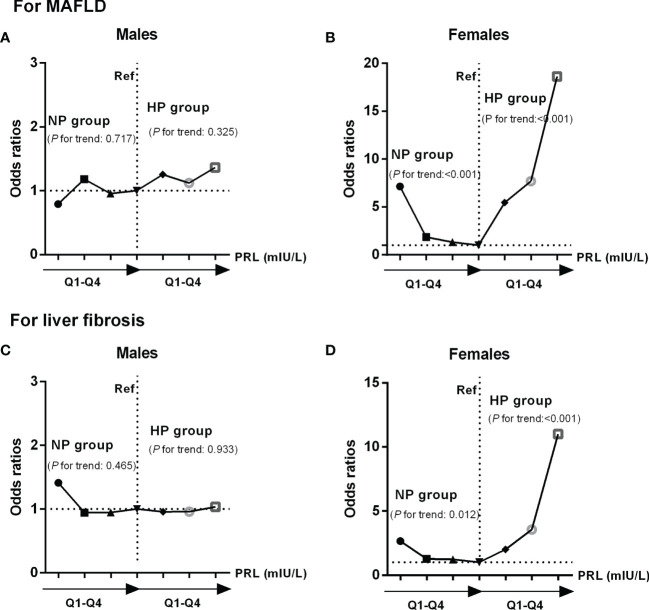
Odds ratios (ORs) and 95% confidence intervals **(CI)** for MAFLD and liver fibrosis according to serum PRL quartiles: results of binary logistic regression analysis. PRL levels were plotted into four quartiles (Q1, Q2, Q3, and Q4) in both genders. Among females, there was a significant J-shaped association between serum PRL and risk of MAFLD (*P*-trend < 0.001) **(B)**, as well as liver fibrosis (*P-*trend < 0.05) across serum PRL quartiles **(D)**. In contrast, such association was not shown in males (*P*-trend > 0.05) **(A, C)**. *P*-values <0.05 were accepted as statistically significant.

In binary logistic regression analysis, we found a significant J-shaped association between serum PRL levels and the risk of MAFLD and hepatic fibrosis in females with T2DM but not in males. Among females, the odds ratios (ORs) and 95% confidence intervals (CI) for MAFLD were 7.135 (2.497, 20.385) in Q1, 1.865 (1.052, 4.041) in Q2, and 1.330 (0.634, 2.791) in Q3 when using the highest quartile (Q4) as reference (*P*-trend < 0.001) in the NP group, while 5.464 (1.627, 18.357) in Q2, 7.690 (2.156, 27.431) in Q3, and 18.619 (4.193, 82.674) in Q4 when comparing with the lowest quartile (Q1) (*P*-trend < 0.001) in the HP group (**Figure 6B**). Furthermore, the ORs (95% CI) for hepatic fibrosis were 2.658 (1.276, 5.538) in Q1, 1.275 (0.620, 2.622) in Q2, and 1.227 (0.594, 2.534) in Q3 as opposed to Q4 (*P*-trend = 0.012) in the NP group, while 2.001 (0.636, 6.286) in Q2, 3.556 (1.095, 11.546) in Q3, and 11.001 (2.817, 42.947) in Q4 as opposed to Q1 (*P*-trend < 0.001) in the HP group (**Figure 6D**). However, the ORs (95% CI) for MAFLD and hepatic fibrosis did not change significantly across serum PRL quartiles in males (**Figures 6A, C**). The results remained significant in females after adjusting for potential confounders (age and BMI involved in model 1; age, BMI, CAP, LSM, ALT, AST, and γ-GT involved in model 2; age, BMI, CAP, LSM, ALT, AST, γ-GT, HDL-C, TG, FPG, E2, TT, and Pg involved in model 3) (**Tables 3** and **4**). The *P*-value for trend was significant in all regression models in females (all *P*-trend < 0.05). When these findings were combined, they revealed a significant J-shaped relationship between serum PRL levels and the risk of MAFLD and liver fibrosis in female but in not male patients with T2DM.

**Table 3:** Multiple-adjusted odds ratios (AORs) and 95% confidence intervals (95% CI) for MAFLD, according to serum PRL quartiles: results of binary logistic regression analysis in different models.

PRL quartiles (pmol/L)	Model 1			Model 2			Model 3		
	*β*	Adjusted OR (95% CI)	*P*	*β*	Adjusted OR (95% CI)	*P*	*β*	Adjusted OR (95% CI)	*P*
Males, NP group									
Q1	−0.172	0.842 (0.308–2.304)	0.737	−0.066	0.936 (0.260–3.363)	0.919	−0.076	0.926 (0.233–3.687)	0.914
Q2	0.086	1.090 (0.365–3.251)	0.877	−0.105	0.900 (0.250–3.241)	0.872	0.138	1.148 (0.285–4.621)	0.846
Q3	0.149	1.161 (0.402–3.347)	0.783	0.489	1.631 (0.463–5.742)	0.446	0.566	1.761 (0.460–6.735)	0.408
Q4		Ref	0.940		Ref	0.800		Ref	0.796
*P-*trend		0.719			0.773			0.519	
Males, HP group									
Q1		Ref	0.626		Ref	0.444		Ref	0.423
Q2	0.264	1.302 (0.467–3.631)	0.614	0.472	1.604 (0.502–5.126)	0.426	0.340	1.406 (0.360–5.494)	0.624
Q3	0.482	1.619 (0.539–4.867)	0.391	0.311	1.365 (0.388–4.801)	0.628	0.151	1.163 (0.261–5.186)	0.843
Q4	−0.240	0.786 (0.273–2.264)	0.656	-0.585	0.557 (0.153–2.027)	0.375	-0.958	0.384 (0.082–1.787)	0.222
*P-*trend		0.811			0.485			0.382	
Females, NP group									
Q1	2.084	8.040 (2.698–23.963)	<0.001	1.702	5.483 (1.601–18.778)	0.007	2.899	18.165 (3.425–96.336)	0.001
Q2	0.876	2.401 (1.042–5.536)	0.040	0.467	1.595 (0.611–4.163)	0.340	0.556	1.784 (0.658–5.002)	0.301
Q3	0.458	1.580 (0.697–3.582)	0.273	0.399	1.490 (0.601–3.692)	0.389	0.479	1.744 (0.608–4.832)	0.255
Q4		Ref	0.002		Ref	0.059		Ref	0.009
*P-*trend		<0.001			0.011			<0.001	
Females, HP group									
Q1		Ref	0.022		Ref	0.030		Ref	0.019
Q2	1.748	5.741 (1.507–21.878)	0.010	1.895	6.651 (1.569–28.192)	0.010	2.407	11.098 (1.819–110.356)	0.004
Q3	1.820	6.172 (1.502–25.363)	0.012	2.268	9.663 (1.763–52.963)	0.009	2.723	15.225 (1.996–116.112)	0.009
Q4	1.952	7.040 (1.340–36.987)	0.021	2.271	9.689 (1.228–76.423)	0.031	2.902	18.211 (2.579–128.568)	0.014
*P-*trend		0.010			0.013			0.020	

PRL levels were plotted into four quartiles (Q1, Q2, Q3, and Q4) according to the serum PRL levels and gender. Model 1: age and BMI were selected. Model 2: age, BMI, CAP, LSM, ALT, AST, and γ-GT were selected. Model 3: age, BMI, CAP, LSM, ALT, AST, γ-GT, HDL-C, TG, FPG, TT, E2, and Pg were selected.

**Table 4:** Multiple-adjusted odds ratios (AORs) and 95% confidence intervals (95% CI) for liver fibrosis, according to serum PRL quartiles: results of binary logistic regression analysis in different models.

PRL quartiles (pmol/L)	Model 1			Model 2			Model 3		
	*β*	Adjusted OR (95% CI)	*P*	*β*	Adjusted OR (95% CI)	*P*	*β*	Adjusted OR (95% CI)	*P*
Males, NP group									
Q1	0.492	1.635 (0.556–4.812)	0.372	0.364	1.439 (0.436–4.748)	0.550	0.741	2.098 (0.447–9.836)	0.347
Q2	−0.268	0.765 (0.249–2.347)	0.639	−0.318	0.728 (0.212–2.498)	0.613	−1.403	0.246 (0.039–1.550)	0.135
Q3	−0.326	0.722 (0.229–2.271)	0.577	−0.142	0.868 (0.249–3.026)	0.824	0.133	1.142 (0.259–5.035)	0.860
Q4		Ref	0.457		Ref	0.694		Ref	0.146
*P-*trend		0.378			0.587			0.644	
Males, HP group									
Q1		Ref	0.349		Ref	0.304		Ref	0.429
Q2	−0.167	0.847 (0.794–6.277)	0.730	0.171	1.186 (0.414–3.402)	0.751	0.512	1.668 (0.517–5.383)	0.392
Q3	−0.698	0.497 (0.678–5.268)	0.179	−0.783	0.457 (0.147–1.415)	0.175	0.709	2.031 (0.580–7.112)	0.268
Q4	−0.803	0.448 (0.386–3.195)	0.128	−0.708	0.492 (0.148–1.634)	0.247	−0.217	0.805 (0.224–2.890)	0.740
*P-*trend		0.080			0.140			0.188	
Females, NP group									
Q1	1.672	5.322 (2.026–13.975)	0.001	1.588	4.893 (1.560–15.347)	0.006	1.814	6.317 (1.685–22.349)	0.006
Q2	0.752	2.120 (1.122–5.467)	0.020	0.685	1.984 (1.179–5.798)	0.011	1.094	2.985 (1.106–9.839)	0.045
Q3	0.615	1.849 (1.005–4.848)	0.011	0.518	1.679 (1.041–4.768)	0.031	0.807	2.242 (0.679–7.406)	0.185
Q4		Ref	0.007		Ref	0.043		Ref	0.049
*P-*trend		0.001			0.005			0.027	
Females, HP group									
Q1		Ref	0.878		Ref	0.572		Ref	0.251
Q2	0.456	1.578 (0.338–3.768)	0.562	1.242	3.462 (0.481–24.903)	0.217	1.013	3.753 (0.262–46.677)	0.283
Q3	0.659	1.933 (0.379–9.863)	0.128	1.445	4.243 (0.471–38.186)	0.197	2.005	7.425 (0.487–13.196)	0.149
Q4	0.210	2.273 (1.340–10.268)	0.044	1.563	4.774 (2.481–28.903)	0.032	2.411	11.151 (1.806–54.266)	0.033
*P-*trend		0.016			0.027			0.030	

PRL levels were plotted into four quartiles (Q1, Q2, Q3, and Q4) according to the serum PRL levels and gender. Model 1: age and BMI were selected. Model 2: age, BMI, CAP, LSM, ALT, AST, and γ-GT were selected. Model 3: age, BMI, CAP, LSM, ALT, AST, γ-GT, HDL-C, TG, FPG, TT, E2, and Pg were selected.

## Discussion

The present study revealed a J-shaped association between serum PRL levels and the risk of MAFLD and liver fibrosis in female participants with T2DM. High-normal serum PRL appeared to be a protective factor for MAFLD and liver fibrosis. Still, hyperprolactinemia may be the risk factor for MAFLD and liver fibrosis. However, such association was not shown in males. These results indicated a significant gender-specific relationship between serum PRL levels, MAFLD, and liver fibrosis among the diabetic population. To our best knowledge, this is the first study to provide new insight into the gender-specific correlation between serum PRL levels and the risk of incident MAFLD and hepatic fibrosis in diabetic patients.

MAFLD, formerly named NAFLD, is a new definition of liver disease associated with known metabolic dysfunction (2) and is well known to coexist with multiple metabolic disorders including obesity, IR, T2DM, dyslipidemia, elevated liver enzymes, inflammatory markers, and hormone imbalance (9, 29–35). It has also been reported to better identify individuals with liver steatosis and significant fibrosis (4). Consistently, we demonstrated that patients with MAFLD had significantly higher BMI, WC, ALT, AST, γ-GT, TG, FINS, HOMA-IR, and CRP levels as well as increased proportions of obesity, hypertension, hyperlipidemia, IR, hepatic steatosis, and fibrosis compared with those without MAFLD in both genders. As for sex hormones, we found significantly higher Pg and lower TT levels in males with MAFLD while higher TT and lower E2 levels in females with MAFLD compared with their counterparts. Based on the fact that various complications accompany MAFLD, we must determine key factors and the potential mechanism in the development and progression of MAFLD among the diabetic population, aiming to provide clinical evidence for its prevention and the treatment of these patients.

PRL, recently identified as a metabolic hormone, has been shown to play a fundamental role in regulating glucolipid metabolism. According to experimental studies, PRL can promote pancreatic β-cell proliferation (17), increase glucose-stimulated insulin secretion and hepatic insulin sensitivity (36), and modulate immune and inflammatory responses (37). The population-based study by Wang et al. (38) reported that a physiologically elevated PRL could protect against diabetes and impaired glucose regulation in both men and women. Ponce et al. (15) found that high-normal PRL levels were associated with improved visceral adipocyte hypertrophy and IR regardless of gender. Strikingly, the relationship between NAFLD and serum PRL has been recently discussed without significant gender difference. In the cross-sectional study by Zhang et al. (16), serum PRL levels were significantly lower in patients with NAFLD than those without NAFLD and negatively associated with the severity of hepatic steatosis in both men and women. Another study by Shao et al. (19) showed that PRL could significantly reduce hepatic TG accumulation in female mice and protect male mice from liver steatosis induced by a high-fat diet. These findings indicate that although the gender-specific role for PRL is clearly well known, the available studies did not observe significant gender difference in the relationship between serum PRL and NAFLD (16, 19). Considering that newly defined MAFLD has not been widely applied in the real world, whether there is gender difference between serum PRL and MAFLD remains unknown. In addition, it is important to notice that different levels of PRL might have different and even opposite effects on metabolic disorders, such as obesity, diabetes, and metabolic syndrome (39–41). Nevertheless, the impact of different levels of PRL on the development and progression of MAFLD is unexplored. Therefore, we conducted this study to investigate the association between serum PRL levels and the risk of MAFLD and liver fibrosis stratified by different PRL subgroups.

We firstly divided patients into NP and HP groups in the present study to test our hypothesis. We found that females with MAFLD had remarkably decreased serum PRL levels in the NP group but increased serum PRL levels in the HP group compared with those without MAFLD, whereas serum PRL levels were unchanged in males. In addition, the proportion of MAFLD had a significantly decreasing trend in the NP group but an increasing trend in the HP group across the serum PRL quartiles among females but not males. After adjusting for potential confounding factors, we observed a J-shaped association between serum PRL and the risk of MAFLD among females but not males, suggesting that high-normal serum PRL may protect against MAFLD while hyperprolactinemia may be a risk factor for MAFLD in females with T2DM but not in males. Our results are dramatically inconsistent with previous research results in which serum PRL acted as a protective factor in developing NAFLD without significant gender difference (16). Importantly, accumulating evidence has validated that hepatic fibrosis is the major adverse outcomes in patients with MAFLD (42). As a result, early and accurate detection of significant fibrosis in MAFLD patients is critical. Recently, FibroScan has been recommended as a useful tool to detect both hepatic steatosis and fibrosis in NAFLD (27). Thus, we assessed liver steatosis and significant fibrosis using FibroScan and explored their correlations with serum PRL in both genders. Likewise, the results showed that females with liver steatosis and significant fibrosis had significantly lower serum PRL levels in the NP group but higher serum PRL levels in the HP group. Also, the proportions of liver steatosis and significant fibrosis presented a remarkably decreasing trend in the NP group but an increasing trend in the HP group with the increment of serum PRL levels among females but not males. Similarly, after multivariate adjustment, the risk for significant fibrosis among females gradually decreased in the NP group. In contrast, it increased in the HP group across the serum PRL quartiles, following a J-shaped curve between serum PRL and significant fibrosis. However, such association was non-significant in males. These results validated our hypothesis that PRL may be relevant to MAFLD and its progression in a gender-specific manner.

Although the exact mechanisms underlying such gender-specific association between PRL, MAFLD, and liver fibrosis remain unclear, several underlying reasons may account for this apparent discrepancy between males and females. To begin with, PRL, as an estrogen-responsive pituitary hormone, has dramatically higher serum levels in women than men, and this difference persists in postmenopausal women compared with men (43). Likewise, our study showed that serum PRL levels were significantly higher in females than in males. Furthermore, serum PRL levels were altered significantly in females with MAFLD, liver steatosis, and significant fibrosis compared with their counterparts and were unchanged among males. The second is that PRL has a more pronounced influence on the inflammatory markers in females compared with males. Low-grade chronic inflammation in the liver is well known to be one of the underlying mechanisms of MAFLD (44). CRP, an acute-phase reactant protein produced primarily by the liver, has been well documented to be associated with MAFLD and used clinically as an inflammatory marker in diagnosing MAFLD (2). Indeed, CRP has been demonstrated to be associated with PRL (45). Several previous studies showed that PRL was associated with increased CRP in individuals with hyperprolactinemia (46) and older adults (47), whereas it was weakly correlated with CRP in male patients (48). Our study found a significant correlation between serum PRL and CRP among females but not males. Thirdly, sex hormones have been well established to be correlated with serum PRL and the presence of MAFLD. A meta-analysis showed that TT levels in men were inversely associated with MAFLD (33). Eguchi et al. (49) demonstrated that testosterone deficiency in men displayed an increased accumulation of visceral adipose tissue and IR, which favor the development of hepatic steatosis.

Furthermore, a long duration of estrogen deficiency was reported to pose a high risk of hepatic fibrosis among postmenopausal women with MAFLD (34), which can be improved by estrogen replacement therapy (50). Conversely, Pg was reported to act in opposition to the favorable effects of estradiol and its effects were blocked by estradiol (35). These results indicate that sex hormones play an important role in the pathogenesis of MAFLD. The present study found that PRL was positively correlated with TT levels among females within the HP group but not with E2 and Pg in males or females. Thus, the gender disparity may be partially explained by the correlation between TT and PRL. Moreover, IR is recognized as a major pathophysiological mechanism of MAFLD. In the present study, we found a significantly positive association between serum PRL and LnFINS and LnHOMA-IR in females within the HP group but not in males. Inconsistently, Ponce et al. demonstrated a significant inverse association between serum PRL and HOMA-IR in obese patients without gender difference (15). Another study revealed that PRL levels were negatively associated with FINS and HOMA-IR in infertile women with PCOS (51).

Furthermore, abnormal liver enzymes often occur in MAFLD patients. In agreement, we found significantly higher ALT, AST, and γ-GT levels in the MAFLD group than in the non-MAFLD group in both genders. However, after adjusting for age and BMI, serum PRL levels were significantly negatively related to ALT in the NP group but positively related to ALT and γ-GT in the HP group among males but not females. In contrast, Yang et al. (52) revealed that serum PRL was negatively associated with AST and ALT in women with PCOS. Another study by Wang et al. (53) showed a significantly negative association between serum PRL and ALT and AST in females but not in males with obesity. Additionally, Garcia-Rizo et al. (54) demonstrated significant correlations of PRL with AST among females but not males. These contradictory results might be explained by different study designs and population, sample size, subgroup analysis, reference range, race, dietary structure, environment, and the genetic makeup of the study population which have been validated as an important predictor in the development of MAFLD (55). Based on these findings, it is reasonable that the relationship between PRL and MAFLD in diabetic patients is gender-specific. However, further studies are needed to validate our findings and elucidate the exact underlying mechanism.

Undeniably, our study has some limitations. One limitation is that hepatic steatosis and fibrosis in our study were assessed using non-invasive methods but not liver biopsy, which is well-known to be the gold standard for diagnosing MAFLD. The reason is that these non-invasive techniques have been validated to be accurate and widely available in the general population (27). Secondly, our retrospective cross-sectional study findings might not reflect the causal relationship between PRL and MAFLD in diabetic patients. Thirdly, unmeasured confounding variables including menstrual cycles, contraceptive use, sexual intercourse, and exercise may exist. In addition, the secretion of PRL is pulsatile, and a single measurement of serum may not be adequate to represent the PRL levels during the whole day. Nonetheless, the pulsatile secretion occurs primarily during the night and is relatively constant during the day (56). Moreover, all subjects in the current study underwent the PRL examination at 8:00 a.m., and hence, the variation of PRL secretion could be avoided. Finally, we defined MAFLD based on the novel international expert consensus in 2020, which has not been widely tested and applied in the real world. Therefore, future studies with many patients whose liver assessment is determined by non-invasive methods and MAFLD is diagnosed based on novel diagnostic criteria are warranted to validate the findings from this study further and identify their underlying mechanism.

## Conclusion

In the present study, we observed a J-shaped association between serum PRL and the risk of MAFLD and liver fibrosis in females with T2DM but not in males, indicating that PRL may be relevant to MAFLD and its progression in a gender-specific manner. Overall, the J-shaped curve is the feature and the highlight of this paper that should be emphasized. It will provide clinicians with a holistic person- and management-centered view of MAFLD. Absolutely, our cross-sectional study might not reflect the causal relationship between PRL and MAFLD, and further studies should focus on delving into the possible mechanism and its clinical significance.

## Data Availability Statement

The raw data supporting the conclusions of this article will be made available by the authors, without undue reservation.

## Ethics Statement

The studies involving human participants were reviewed and approved by Ethics Committee of the Shanghai Tenth People’s Hospital, Tongji University in China. The patients/participants provided their written informed consent to participate in this study.

## Author Contributions

SQ, LB, CZ, and HM contributed to the conception and design of the study. CZ, HM, DH, GL, JG, MC, and HY contributed to the data collection, statistical analysis, and interpretation of the data. CZ and HM organized the data and wrote the first draft of the manuscript. LB and SQ reviewed and revised the manuscript critically for important intellectual content. All authors contributed to the article and approved the submitted version.

## Funding

This project was financially supported by grants from the National Key R&D Program of China (No. 2018YFC1314100), the Shanghai Committee of Science and Technology of China (Nos. 18411951803 and 17DZ1910603), National Natural Science Foundation of China (No. 81970677), the Shanghai Pujiang Program (Nos. 2019PJD040 and 2018PJD038), and the New Exploration of Blood Glucose Management Mode in Patients with Diabetes Mellitus in Chongming Area of Shanghai (No. CKY2018-19).

## Conflict of Interest

The authors declare that the research was conducted in the absence of any commercial or financial relationships that could be construed as a potential conflict of interest.

## Publisher’s Note

All claims expressed in this article are solely those of the authors and do not necessarily represent those of their affiliated organizations, or those of the publisher, the editors and the reviewers. Any product that may be evaluated in this article, or claim that may be made by its manufacturer, is not guaranteed or endorsed by the publisher.
